# Adoption of recommended practices and basic technologies in a low-income setting

**DOI:** 10.1136/archdischild-2013-305561

**Published:** 2014-01-30

**Authors:** Mike English, David Gathara, Stephen Mwinga, Philip Ayieko, Charles Opondo, Jalemba Aluvaala, Elesban Kihuba, Paul Mwaniki, Fred Were, Grace Irimu, Aggrey Wasunna, Wycliffe Mogoa, Rachel Nyamai

**Affiliations:** 1 KEMRI-Wellcome Trust Research Programme, Nairobi, Kenya; 2 Nuffield Department of Medicine & Department of Paediatrics, University of Oxford, UK; 3 Health Services, Implementation Research and Clinical Excellence (SIRCLE) Collaboration, KEMRI-Wellcome Trust Research Programme, Nairobi, Kenya; 4 Ministry of Health, Nairobi, Kenya; 5 Department of Paediatrics and Child Health, University of Nairobi, Nairobi, Kenya

**Keywords:** Health services research, Tropical Paediatrics

## Abstract

**Objective:**

In global health considerable attention is focused on the search for innovations; however, reports tracking their adoption in routine hospital settings from low-income countries are absent.

**Design and setting:**

We used data collected on a consistent panel of indicators during four separate cross-sectional, hospital surveys in Kenya to track changes over a period of 11 years (2002–2012).

**Main outcome measures:**

Basic resource availability, use of diagnostics and uptake of recommended practices.

**Results:**

There appeared little change in availability of a panel of 28 basic resources (median 71% in 2002 to 82% in 2012) although availability of specific feeds for severe malnutrition and vitamin K improved. Use of blood glucose and HIV testing increased but remained inappropriately low throughout. Commonly (malaria) and uncommonly (lumbar puncture) performed diagnostic tests frequently failed to inform practice while pulse oximetry, a simple and cheap technology, was rarely available even in 2012. However, increasing adherence to prescribing guidance occurred during a period from 2006 to 2012 in which efforts were made to disseminate guidelines.

**Conclusions:**

Findings suggest changes in clinical practices possibly linked to dissemination of guidelines at reasonable scale. However, full availability of basic resources was not attained and major gaps likely exist between the potential and actual impacts of simple diagnostics and technologies representing problems with availability, adoption and successful utilisation. These findings are relevant to debates on scaling up in low-income settings and to those developing novel therapeutic or diagnostic interventions.

**What is already known**

- District hospitals are an important part of the primary healthcare system but often provide poor quality of care in low-income settings.
- Considerable effort is devoted to developing new recommendations and technologies to improve hospital care but adoption in low-income settings is rarely explored.
- Adoption of innovations spanning new recommendations and technologies has proceeded slowly in developed countries.

**What this study adds**

- No change was followed by slow change in clinical practices over 6 years in Kenyan hospitals, possibly linked to dissemination of guidelines at scale.
- Major gaps exist between potential and actual impacts of simple diagnostics and technologies highlighting problems with availability, adoption and successful utilisation.
- Data to support routine monitoring of hospital care, outcomes and uptake of innovations are completely inadequate but much needed

## Introduction

The Millennium Development Goals (MDGs) helped bring international attention to the need to reduce child mortality globally. Although subsequent reports suggest progress this is least marked in many African countries where changes at national scale in coverage of essential primary healthcare interventions has often been limited.^1^ In Kenya although child mortality has fallen from 98/1000 to 73/1000 live births between 1990 and 2011, progress is insufficient to meet MDG targets with 40% of under 5 mortality now attributable to neonatal mortality.^2^ In common with other settings increasing recognition is being given to the need for broader health system strengthening to improve the health of its 6.8 million children aged under 5, 107 000 of whom die annually. The ability of the first referral (district hospital) level to support child survival seemed first to gain attention after publication in 2000 of the work of a WHO team.^3^ This work indicated limited capacity to provide appropriate interventions and very poor adoption of recommended practices and technologies in district hospitals, findings subsequently substantiated by others in a number of settings.^4–6^ Yet, over the past two decades considerable resources have been devoted to the development of new therapeutic and diagnostic technologies. Indeed, these themes are common across four of the five research challenges defined by The Wellcome Trust as major goals while for The Bill and Melinda Gates Foundation ‘All strategies underscore the role of technology’.^7^ ^8^ As district hospitals typically serve as hubs linked to multiple primary care facilities and are likely to have important roles in formal and informal knowledge networks in rural areas, we suggest they may be a useful general barometer of the routine adoption of innovations in health systems.

Here, therefore, we use observations collected over an 11-year period to track whether there have been any improvements in essential paediatric hospital services and track the adoption of basic national and international guideline recommendations. Importantly WHO and national guidelines for management of the seriously ill child with common diseases have been available since 2000 in aggregated form and earlier in disease-specific programme guidance and provide common, basic standards for assessments across this period. In Kenya specific efforts to develop and promote these guidelines in a simple, cheap format began in 2006^9^ with distribution of 10 000 copies in 2007/2008 and 12 000 copies in 2010/2011.^10^ During this period, a training programme to support use of guidelines also reached over 1500 clinicians and nurses (less than 10% of the public sector health workforce) and was integrated into undergraduate and postgraduate teaching in the largest medical school from 2008.^10^ Although there have been many other health interventions over the decade examined, we are not aware of any other specific, major efforts targeting implementation of such inpatient paediatric guidelines. In describing the adoption of basic diagnostics, technologies and practice recommendations, it is not our purpose to draw any inference on the effectiveness of Kenyan efforts to popularise guidelines. Instead such efforts are part of the context from which data are drawn that we use to pose questions about the adequacy of progress and, more generally, our ability to determine whether innovations that should be efficacious are available and successfully used in routine settings.

## Methods

We make use of data collected as part of different studies using similar methodology and tools (described in detail in^4^ ^11–13^) from a period spanning 2002 to 2012 with the surveys and available data summarised in table 1. Each survey received appropriate ethical and institutional approval at the time it was undertaken. In brief they involved 14 hospitals and examination of 641 case records in 2002 and 8, 17 and 22 hospitals and 2135, 952 and 1298 case records divided reasonably evenly across sites in 2006, 2009 and 2012, respectively. Core indicators remained the same across surveys including the ability to offer key diagnostic tests for: malaria, HIV rapid testing, haemoglobin, blood glucose and lumbar puncture for meningitis; together with the availability of two additional diagnostic tools, blood-based total bilirubin measurement (for the assessment of neonatal jaundice) and pulse oximetry for paediatric admissions. The availability of key therapeutic or practice resources for managing sick children or newborns was also assessed by direct observation of survey team leaders and included: the proportion of 28 key resources (9 items of equipment and 19 consumables) available at the time of survey and the specific availability of: paediatric and neonatal bag valve mask devices, specifically formulated milk feeds for severe malnutrition (F75/F100), vitamin K for neonatal prophylaxis and phototherapy equipment for neonatal jaundice.

**Table 1:** Basic characteristics of four hospital surveys conducted over the period 2002 to 2012 and definitions of patient level indicators

	2002^5^	2006^11^	2009^12^	2012^13^
Number of hospitals	14	8	17	22
Hospital selection	Purposeful	Purposeful	Purposeful	Purposeful
Total cases examined	641	2135	952	1298
Case selection process	Convenience sample of children on ward during survey period with acute non-surgical illnesses	Random sample of paediatric inpatient records over six months period prior to survey with acute non-surgical illnesses	Convenience sample of paediatric inpatient records from most recent discharges with malaria, pneumonia and diarrhoea	Convenience sample of paediatric inpatient records from most recent discharges with acute non-surgical illnesses

*Indicator calculations for children with an admission diagnosis of an acute, non-surgical illness*	
Use of malaria testing	Proportion with an admission diagnosis of malaria for whom a malaria test (blood slide examination or rapid test) is ordered to substantiate diagnosis
Use of HIV testing (at least rapid testing)	Proportion for whom an HIV test is requested (typically a first rapid test). Routine HIV testing for all admissions has been recommended in Kenya as part of formerly diagnostic testing and counselling and latterly provider initiated testing and counselling
Use of haemoglobin measurement	Proportion with clinically documented pallor for whom a measured haemoglobin is requested as a standalone test or as part of a full blood count using any available laboratory method
Use of blood glucose measurement	Proportion of admissions for whom a blood glucose measurement is requested/documented either as a rapid bedside test or as a formal laboratory test using any available laboratory method
Use of lumbar puncture to identify meningitis	Proportion of all admissions for whom a lumbar puncture is requested/documented by the admitting clinical team to rule out/rule in meningitis
*Indicator calculations among children with specific admission diagnoses*	
Use of a quinine loading dose	Proportion with an admission diagnosis of malaria for whom a quinine prescription began with a loading dose to initiate therapy
Use of once daily gentamicin	Proportion of children prescribed gentamicin in whom therapy was based on the recommended once daily regimen
Use of a specific fluid plan for children with severe dehydration	Proportion of children given iv fluids for severe dehydration in whom the nature, volume and duration of initial fluids prescribed matched national and international recommendations

Finally, we examined use of the five important diagnostic tests (table 1, data from 2002, 2006 and 2012) and adherence to one clinical practice guideline, unchanged since 2000 or earlier, for each of malaria, pneumonia and diarrhoea (data from 2002, 2006, 2009 and 2012). These were: use of a quinine loading dose, once daily gentamicin (no data for 2009) and a correct fluid plan for children with severe dehydration (table 1). As paediatric prescribing is based on weight we also tracked whether clinicians documented this in the case record.

### Statistical analysis

We present simple frequencies for indicators measured at hospital level and use the median of hospital-specific proportions or simple aggregate proportions as a point estimate for indicators based on evaluation of multiple cases from each survey. As surveys did not employ random sampling of facilities and records we do not provide estimates of precision around these point estimates. Neither is further statistical testing of change or trends appropriate given the variation in facilities sampled and sampling approaches. We accept the limitations of this approach and suggest a very cautious interpretation of results but use these crude data to draw basic lessons now while highlighting the need for better data in the future to understand adoption of innovations.

## Results

### Basic resources

The median availability of a set of 28 very basic resources in hospitals was 20/28 items (71%, range 7/28 to 24/28), 21.5/28 (77%, range 14/28 to 25/28) and 23/28 (82%, range 15/28 to 28/28) in 2002, 2006 and 2012, respectively. Although the nature of missing resources varied across place and time, there seemed consistent difficulties in access to antistaphylococcal penicillins and phenobarbitone injection, the latter being the mainstay of treatment for prolonged convulsions in children and the only therapy routinely recommended for neonatal convulsions in Kenya. The specific availability of feeds for children with severe malnutrition and resuscitation equipment increased from 2006 over the period examined (table 2). Some progress was apparent for availability of vitamin K to prevent haemorrhagic disease of the newborn with additional data suggesting parallel improvements in use; 19/189 (10%) and 843/1213 (70%) eligible newborns received vitamin K in 2006 and 2012, respectively.

**Table 2:** Resources for providing effective paediatric and neonatal care in Kenyan district hospitals illustrated using data on four key indicators evaluated in the period 2002–2012

	2002	2006	2009	2012
*Availability of key therapeutic resources*				
F75/F100 for children with severe malnutrition	0/14 (0%)	2/8 (25%)	16/17 (94%)	22/22 (100%)
Vitamin K for neonatal prophylaxis	2/14 (14%)	0/8 (0%)	12/17 (71%)	18/22 (82%)
Appropriate bag-valve-mask devices in paediatric areas	0/14 (0%)	6/8 (75%)	12/17 (71%)	22/22 (100%)
Availability of working phototherapy device	8/14 (57%)	5/8 (63%)	14/17 (82%)	19/21* (90%)
*Availability of basic diagnostic tests*				
Pulse oximetry	0/14 (0%)	0/8 (0%)	0/17 (0%)	3/22 (14%)
Malaria slide or other diagnostic	14/14 (100%)	8/8 (100%)	17/17 (100%)	22/22 (100%)
Haemoglobin measurement	14/14 (100%)	8/8 (100%)	17/17 (100%)	22/22 (100%)
Blood glucose testing	14/14 (100%)	8/8 (100%)	17/17 (100%)	22/22 (100%)
Microscopy, Gram stain and culture for CSF	7/14 (50%)	8/8 (100%)	17/17 (100%)	22/22 (100%)
Bilirubin measure (blood based)	6/14 (43%)	3/8 (38%)	16/17 (94%)	19/22 (86%)
HIV testing (rapid)	14/14 (100%)	8/8 (100%)	17/17 (100%)	22/22 (100%)
*Availability of information resources*				
Paediatric guidelines available	0/14 (0%)	0/8 (0%)	14/17 (83%)	16/22 (73%)
Newborns given medical record	0/14 (0%)	0/8 (0%)	–†	20/22 (91%)

Results expressed as number of hospitals in which resource was available over number of hospitals evaluated (%).

*Data missing for one hospital.

†Data not collected in survey.

### Basic diagnostics

**Figure 1:**
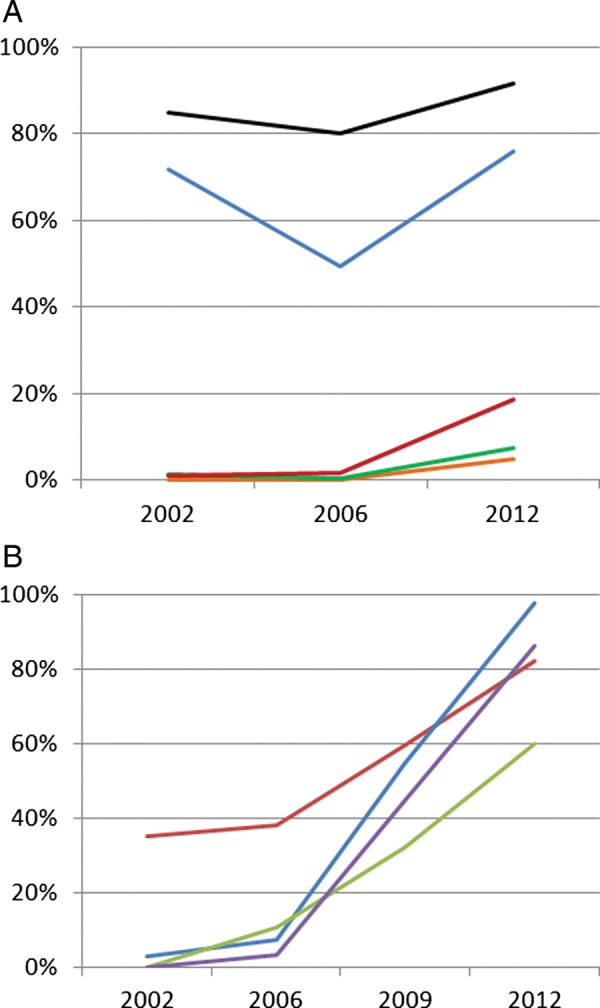
Panel A: Use of diagnostic tests in children admitted to hospital. Y-axis represents proportion of eligible population with test ordered (see table 1 for definitions), X-axis represents year of survey. Malaria blood slide—black line; Haemoglobin—blue line; HIV test—red line; lumbar puncture—green line; blood glucose—orange line. Panel B: Clinical practices indicating adherence to guidelines. Y-axis represents proportion of eligible population with treatment prescribed consistent with guideline (see table 1 for definitions), X-axis represents year of survey. Weight documented in medical record—red line; quinine loading dose—blue line; once daily gentamicin—purple line (no data for 2009); correct intravenous fluid regimen—green line.

All hospitals at all time points had the ability to offer four key diagnostic tests: malaria blood slide, haemoglobin measurement, blood glucose measurement and HIV testing (table 2). The ability to examine cerebrospinal fluid after lumbar puncture or measure total bilirubin in blood improved over time (table 2, note: transcutaneous bilirubin measurement is not available at all). Pulse oximetry was not available in any hospital until 2012 and then in only 3/22 sites (table 2). Laboratory testing of children with a clinical diagnosis of malaria was common at all time points (84% to 91% cases) while requests for a haemoglobin measurement in children documented to have pallor were somewhat less common (49% to 76%) (figure 1, Panel A).

The proportion of paediatric admissions for whom blood glucose, lumbar puncture or HIV testing was requested increased after 2006. In the case of HIV testing it has been national policy since at least 2004 to test all paediatric admissions. Requesting testing in less than 20% of admissions overall in 2012 therefore still appears inappropriately low. Clinical signs are poor at identifying hypoglycaemic children, testing is cheap, 10% or more of paediatric admissions may be hypoglycaemic and it is a clear risk factor for death.^14^ As national guidelines recommend testing of all severely ill children testing fewer than 10% of admissions also still seems inappropriately low. Meningitis is uncommon but should be ruled out by testing at risk children. Requesting lumbar puncture increased from a rate of 1.2% (in 2002) to 7.4% of all admissions in 2012, a period after introduction of *Haemophilus influenzae* Type B (Hib) (in 2001) and pneumococcal vaccines (in 2011). A correct rate of lumbar puncture is not possible to define but in most Kenyan hospitals at least 10% of children are admitted with fever complicated by convulsions (unpublished data). However, anecdotal experience suggests few of the requested lumbar punctures are actually performed with results of tests rarely found in records.

### Changes in prescribing practice

Availability of paediatric guidelines improved (table 2) after specific efforts to produce and disseminate a simple, low-cost version. There was little evidence of adherence to guidelines between 2002 and 2006. However, use of a quinine loading dose, once daily gentamicin and correctness of fluid plans for children with severe dehydration all improved between 2006 and 2012 (figure 1, Panel B), as did documentation of patient weight, although there remains considerable room for further improvement.

### Service delivery information

Recognising sick newborns as admissions has improved (table 2). However, in 2009 and 2012 data quality were too poor to calculate hospital-based neonatal mortality rates and data on stillbirths were generally missing or unreliable. Even for paediatric mortality age stratified estimates in 2009 and 2012 could only be estimated for 7/17 and 8/22 hospitals, respectively. When attempted in 2009 a 12–16-fold difference in reported inpatient infant mortality rates was observed raising doubts about the credibility of such data even where available.^12^ ^13^

## Discussion

One in five basic resource items for providing care to seriously ill children and newborns were typically not available over the period of study but improvements were seen for some key areas (eg, feeds for malnutrition and vitamin K). Compliance with clinical prescribing recommendations in three areas improved from below 20% to above 60% over a period of 6 years, a period during which guidelines were widely disseminated in a simple cheap format and knowledge-based and skills-based training was expanded through in-service and preservice training. Over the same period there was some improvement in the use of basic diagnostics but this was much more limited.

Alternative explanations for any improvement observed might include simple secular changes in hospital services. Although government expenditure on health did rise in absolute terms over this period, there was no significant increase year on year relative to population growth and inflation.^15^ Government partner spending did dramatically increase. For example grants to Kenya from the GFATM have amounted to $2655 million since 2000 and USAID report allocating approximately 84% of their country budgets to health, amounting to sums of over $330 million per year in 2010 and 2011.^16^ ^17^ Much of this funding has been directed to supporting HIV, malaria or TB specific primary care services; however, there has been little direct support for inpatient paediatric hospital care. Perhaps indicative of this one of the simplest and cheapest bedside diagnostic tests, pulse oximetry, remained largely unavailable over the entire period while CD4 analysers are now present in most hospitals in Kenya.

While it is hard to discern clearly which factors contributed to improvements we believe greater knowledge and thus better advocacy may have been important in addressing specific resource gaps (such as appropriate sized resuscitation devices).^18^ We believe access to basic guidelines, formal and informal knowledge sharing mechanisms, engagement with the national paediatric association and a resulting change in professional expectations supported greater attention to paediatric prescribing in particular. We contend that multiple factors contributed to persistent relatively poor use of basic diagnostics including: financial constraints, lack of trust from clinicians in timely access to quality results (reducing requests), role ambiguity in the case of HIV testing and reluctance to pass costs of testing on to patients.^19^

Our findings are important for a number of reasons. First, if we wish to see changes at scale major, sustained and adequately resourced efforts will be needed. These should comprise wide, ‘whole system’ interventions if our aim is to improve overall service delivery.^20^ ^21^ Second, although considerable funding is being devoted to the development of novel diagnostics, our data suggest that even when cheap diagnostic technologies are ‘brought to market’ they will not necessarily become available or be used. To achieve this we must also solve implementation challenges that are as much to do with the complexity of the political, social and professional systems as their cost and availability.^22^ ^23^ Those supporting the development of such innovations would perhaps do well to try and understand these challenges and explore, for example, why pulse oximetry has failed to become a standard of care in African hospitals.

It is clear that the data presented here are limited in coverage, scope and quantity. We acknowledge this and accept that findings should be interpreted cautiously. However, an additional purpose of this report is to highlight major inadequacies in information systems. These are not providing reliable routine data on outcomes of hospital care and provide no real insight into the process of care during service delivery. Yet both are critical to an understanding of health system performance. As we invest in new innovations there appears a clear case, therefore, to invest in appropriate information systems and analyses to assess their actual impact.

In conclusion, our data suggest slow but steady progress in the adoption of signal clinical practices relevant to management of common serious illnesses among paediatric admissions in a setting where there have been efforts to improve such practices. However, a decade after highlighting inadequacies in basic resources these are not uniformly available while adoption and use of diagnostics and technologies often remains very limited. Unfortunately, we are not aware of reports from other settings with which we can compare our findings, something that may reflect the paucity of health services data from low-income countries. As we approach and move beyond 2015 continued improvements in child survival we believe will require broadly effective health systems and health information systems. We suggest that the performance of district hospitals in delivering essential services should be an integral part of national monitoring and system strengthening.
